# Design, Metallurgical Features, and Mechanical Behaviour of NiTi Endodontic Instruments from Five Different Heat-Treated Rotary Systems

**DOI:** 10.3390/ma15031009

**Published:** 2022-01-28

**Authors:** Jorge N. R. Martins, Emmanuel J. N. L. Silva, Duarte Marques, Mário Rito Pereira, Victor T. L. Vieira, Sofia Arantes-Oliveira, Rui F. Martins, Francisco Braz Fernandes, Marco Versiani

**Affiliations:** 1 Faculdade de Medicina Dentária, Universidade de Lisboa, 1600-277 Lisboa, Portugal; duartemd@yahoo.co.uk (D.M.); mariorp@me.com (M.R.P.); sofiaaol@campus.ul.pt (S.A.-O.); 2 Grupo de Investigação em Bioquimica e Biologia Oral, Unidade de Investigação em Ciências Orais e Biomédicas (UICOB), 1600-277 Lisboa, Portugal; 3 Centro de Estudo de Medicina Dentária Baseada na Evidência (CEMDBE), 1600-277 Lisboa, Portugal; 4 Department of Endodontics, Grande Rio University (UNIGRANRIO), Rio de Janeiro 21210-623, Brazil; nogueiraemmanuel@hotmail.com (E.J.N.L.S.); victortalarico@gmail.com (V.T.L.V.); 5 Department of Endodontics, Fluminense Federal University, Niterio, Rio de Janeiro 24220-900, Brazil; 6 LIBPhys-FCT UID/FIS/04559/2013, 1600-277 Lisboa, Portugal; 7 BIOMAT, Laboratório de Biomateriais, Unidade de Investigação em Ciências Orais e Biomédicas (UICOB), 1600-277 Lisboa, Portugal; 8 UNIDEMI, Department of Mechanical and Industrial Engineering, NOVA School of Science and Technology, Universidade NOVA de Lisboa, 2829-516 Caparica, Portugal; rfspm@fct.unl.pt; 9 CENIMAT/I3N, Department of Materials Science, NOVA School of Science and Technology, Universidade NOVA de Lisboa, 2829-516 Caparica, Portugal; fbf@fct.unl.pt; 10 Dental Specialty Center, Brazilian Military Police, Belo Horizonte 30350-190, Brazil; marcoversiani@yahoo.com

**Keywords:** bending load, cyclic fatigue, endodontics, microhardness, rotary system, torsional resistance

## Abstract

The current study aimed to compare the F1 endodontic instruments from five different heat-treated rotary systems regarding their design, metallurgical properties, and mechanical performance. Five F1 root canal shaping instruments (ProTaper Gold [PTG], Premium Taper Gold, Go-Taper Flex, EdgeTaper Platinum, and Super Files Blue)—plus, a conventional ProTaper Universal (PTU)—which were evaluated regarding their design, nickel/titanium ratio, phase transformation temperatures, microhardness, cyclic fatigue, and torsional and bending strengths. Mood's median test was used for the statistical comparison with a significance set at 5%. The instruments were similar regarding the nickel/titanium ratio and overall design. Go-Taper Flex had the closest transformation temperatures to PTG. PTU and Go-Taper Flex had the highest microhardness (408.3 and 410.5 HVN). The time to fracture of Super Files Blue was three and seven times higher than PTG and PTU, respectively. No difference was observed in the maximum torque to fracture among PTG (1.30 N·cm) and the other systems, except for the Premium Taper Gold (1.05 N·cm) and Go-Taper Flex (1.10 N·cm). Significantly lower bending loads than PTG (269.2 gf) were observed for the EdgeTaper Platinum (158.3 gf) and Premium Taper Gold (103.5 gf) instruments. Super Files Blue outperformed PTG in the cyclic fatigue test, while EdgeTaper Platinum and Premium Taper Gold were more flexible. Premium Taper Gold and Go-Taper Flex showed lower torsional strength.

## 1. Introduction

In dental clinical practice, endodontic instrument fracture occurs in a dynamic mechanism that may result from flexural and torsional stresses. Over the years, the improvement of nickel–titanium (NiTi) instruments have been one of the main goals of manufacturers for a more effective and safe mechanical preparation of the root canal system. These approaches include changing the instrument’s taper and cross-sectional design over the length of the cutting blades, as well as the enhancement of the manufacturing process by using new alloys or thermomechanical treatments [1,2]. Heat treatment of NiTi alloy has been frequently used to optimize its microstructure and transformation behavior, increasing its shaping memory characteristics [3,4] and, consequently, improving its mechanical properties [5,6]. For instance, although the ProTaper Gold (Dentsply Sirona, Ballaigues, Switzerland) and ProTaper Universal (Dentsply Maillefer, Ballaigues, Switzerland) instruments share the same geometries, with a convex triangular cross section and progressive taper, the thermomechanical treatment of the PTG alloy increased its flexibility and resistance to cyclic fatigue [5,7]. The terms M-Wire (Dentsply Maillefer), CM Wire (Coltène, Allstetten, Switzerland), MaxWire (FKG Dentaire, La Chaux-de-Fonds, Switzerland), FireWire (EdgeEndo, Johnson City, TN, USA), R-phase wire (SybronEndo, Orange, CA, USA), or Gold and Blue heat treatments (Dentsply Maillefer) are examples of designations given to proprietary advanced metal alloys or heat treatments [4]. 

Recently, several companies worldwide have been producing or distributing heat-treated NiTi endodontic instruments for the mechanical preparation of root canals. Some of them are alternative systems that present characteristics that are quite similar to the original brands [8]. Most of these alternative preparations systems have been made available to clinicians and are currently marketed without proper evidence of their overall performance, intrinsic characteristics, or safety. The multimethod research, or “mixed methods research”, focuses on combining qualitative and quantitative methods and recognizing both the strengths and weaknesses of each one, while also aiming for a superior strengths compensation, which ultimately may lead to more credible results and reliable answers [9]. 

The heat-treated ProTaper Gold (PTG) and conventional ProTaper Universal (PTU) are among the most used NiTi rotary root canal preparation systems in endodontics and are both made of three shaping (named as Sx, S1, and S2) and three finishing files (F1, F2, and F3). The F1 file is the initial finishing instrument and the first one with the objective of actively enlarging the root canal apical area. Due to that reason, it is an instrument with special strength needs in that particular apical area. Therefore, the present study aimed to compare the F1 root canal shaping instruments from premium brands PTU, PTG, and four other NiTi heat-treated replica-like rotary systems throughout a multimethod research perspective by assessing the instrument’s design, metallurgical properties, microhardness, and mechanical performance. The null hypotheses to be tested were that there were no differences amongst the tested systems concerning their (i) design, (ii) metallurgical characterization, (iii) surface microhardness, and (iv) mechanical performance.

## 2. Materials and Methods

**Figure 1:**
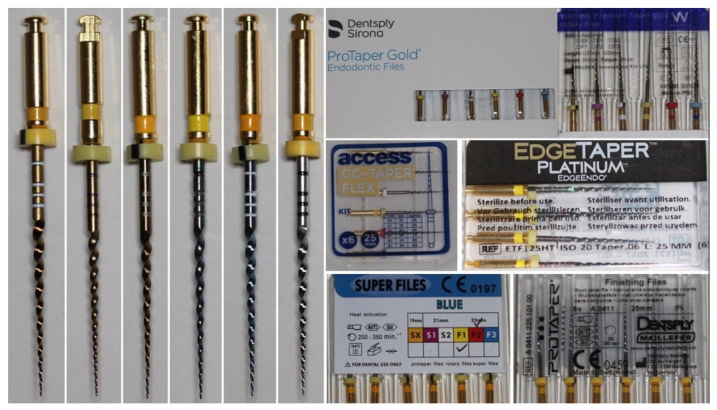
Macroscopic images of the 6 tested instruments (from left to right: ProTaper Gold, Premium Taper Gold, Go-Taper Flex, EdgeTaper Platinum, Super Files Blue and ProTaper Universal) with their respective labelled packing boxes.

Five F1 (B1) instruments (PTG, Premium Taper Gold, Go-Taper Flex [whose instruments are designated as B1], EdgeTaper Platinum, and Super Files Blue) (Table 1, Figure 1, n = 50 per group) were evaluated regarding their design, metallurgical characteristics, surface microhardness, and mechanical performance. A well-known F1 instrument made of a conventional NiTi alloy (PTU system) was used as control. A total of 300 instruments were tested. 

### 2.1. Instruments’ Design

Six instruments from each system were randomly selected and examined under a stereomicroscopic visual inspection through a dental operating microscope (Opmi Pico, Carl Zeiss Surgical, Munich, Germany) at ×3.4 and ×13.6 magnifications for the following characteristics: (a) number of active blades (in units); (b) helical angle of the active blade based on the average measurements of the six most coronal angles assessed in triplicate; (c) distance (in mm) from the instrument’s tip to the three measuring lines (18, 20, and 22 mm) at their non-cutting part by using a digital caliper with a resolution of 0.01 mm (Mitutoyo, Aurora, IL, USA). Discrepancies were considered significant when values were higher than 0.1 mm from the reference line. This process was repeated three times and a mean reading was recorded; and (d) identification of major defects or deformations (missing, twisted, or distorted blades). Then the same instruments were evaluated at ×100 magnification under a conventional scanning electron microscopy (SEM) (S-2400, Hitachi, Tokyo, Japan) regarding: (a) spiral design at the middle portion of the active part (symmetrical or asymmetrical); (b) tip (active or non-active); (c) cross-sectional design; (d) presence of surface marks (machined production process); and (e) minor manufacturer defects or deformations. 

### 2.2. Metallurgical Characterization

Semi-quantitative elemental analysis was performed by energy-dispersive X-ray spectroscopy and scanning-electron microscopy (EDS/SEM), while differential scanning calorimetry (DSC) assessed the transformation characteristics of the instruments. The EDS/SEM analysis was conducted on a 500 µm × 400 µm surface area of 3 instruments from each system at a 25-mm distance through a scanning electron microscope (Zeiss DSM 962; Carl Zeiss Microscopy GmbH, Munich, Germany) set at 20 kV and 3.1 A, and connected to a EDS detector (Inca X-act; Oxford Instruments NanoAnalysis, Abingdon, United Kingdom) by using a dedicated software with ZAF correction (Microanalysis Suite V4.14; Oxford Instruments NanoAnalysis, Abingdon, UK). The DSC analysis was achieved by testing two instruments from each system (DSC 204 F1 Phoenix; Netzsch-Gerätebau GmbH, Selb, Germany) according to guidelines from the American Society for Testing and Materials [10]. A 3 to 5-mm fragment weighing 7 to 10 mg was cut from the coronal active portion of the instruments and submitted to a chemical etching. The prepared fragments were then mounted in an aluminum pan, while an empty pan served as a control. Each thermal cycle was performed under a gaseous nitrogen (N_2_) atmosphere with temperatures ranging from 150 °C to −150 °C. The Netzsch Proteus Thermal Analysis software (Netzsch-Gerätebau GmbH) was used to create the DSC charts that allowed the visual analysis of the transformation temperatures. The second test was done to confirm the results of the first one.

### 2.3. Microhardness Test

The instruments’ microhardness tests were conducted in a Vickers Hardness tester (Duramin; Struers Inc., Cleveland, OH, USA) by making five indentations on each instrument’s surface. The sample size determination had been taken into consideration for the indentations of the first file and the systems that were presenting a higher discrepancy in their results. Considering an effect size of 168.4 and a standard deviation of 91.9 (80% power and alpha of 0.05) (PTU vs Premium Taper Gold), a total of six indentations would be needed. Since the calculation did not take into consideration the other 4 groups, a total of 15 indentations (3 instruments overall) were chosen. Each instrument was prepared according to ASTM standards and stabilized in an acrylic support, and the diamond penetrator was set to perform a 100 gram/force (gf) press load for 15 s [11]. The evaluation was done using a 40× objective. 

### 2.4. Mechanical Tests

Cyclic fatigue, torsional, and bending resistance tests were used to evaluate the mechanical performance of the selected instruments. A sample size calculation with 80% power and an alpha of 0.05 was performed for each test based on the highest difference obtained in the six initial measurements between two systems. 

A total of seven instruments per group was determined for the time to fracture test based on an effect size of 298.5 ± 166.9 (PTU vs Super Files Blue). For the maximum torque and angle of the rotation parameters, sample sizes of seven and six instruments were determined considering the effect sizes of 0.45 ± 0.27 (Super Files Blue vs Go-Taper Flex) and 410.5 ± 226.6 (Go-Taper Flex vs Premium Taper Gold), respectively, while six instruments were required for the maximum load test based on an effect size of 287.0 ± 150.0 (PTU vs Premium Taper Gold). Therefore, a total of 12 instruments per group was defined for each dependent variable. Previous to the tests however, each instrument was submitted to a stereomicroscopic visual inspection under ×13.6 magnification to detect any deformations or defects that would exclude them. No deformities were observed. 

For the cyclic fatigue test, a stainless-steel custom-made tube model apparatus was used according to a previous study [8]. The instruments from each system were activated at a headpiece static position with a 6:1 reduction hand-piece (Sirona Dental Systems GmbH, Bensheim, Germany) powered by a torque-controlled motor (VDW Silver; VDW GmbH, Munich, Germany) in a continuous clockwise rotation mode at 300 rpm and 2.0 N torque, using glycerin as lubricant. The tests were performed on an 86 degrees and 6 mm radius artificial canal curvature with a 1.4 mm inner diameter. The time to fracture (in seconds) was determined when the instrument fracture was detected, visually and/or audibly, using a digital chronometer. The size of the fractured segments was recorded in millimeters using a digital caliper (Mitutoyo) only for the experimental control. The torsional and bending resistance tests were performed according to the ISO 3630-1:2019 specification [12]. For the static torsion test, instruments were clamped in their apical at 3 mm and rotated clockwise at a constant pace of 2 rotations per minute until rupture. The maximum torque (in N·cm) and angle of rotation (in degrees) prior to fracture were determined by a torsimeter (TT100 Odeme Dental Research, Luzerna, Santa Catarina, Brazil). For the bending test, the instruments were mounted by their grip in the file holder of the motor at a 45° position in relation to the floor plane, while having their apical at 3 mm attached to a wire linked to a universal testing machine (Instron EMIC DL-200 MF, São José dos Pinhais, Brazil) using a 20 N load-cell at 15 mm/min constant speed, until a 45° displacement occurs. The maximum load required to promote the displacement was recorded in gf. 

### 2.5. Statistical Analysis 

The results of all the tested variables showed a non-Gaussian distribution (Shapiro–Wilk test, *p* < 0.05) and statistical comparisons were performed among groups using the nonparametric Mood’s median test with a significance level set at 0.05 (SPSS v22.0 for Windows; SPSS Inc., Chicago, IL, USA).

## 3. Results

### 3.1. Instruments’ Design 

**Figure 2:**
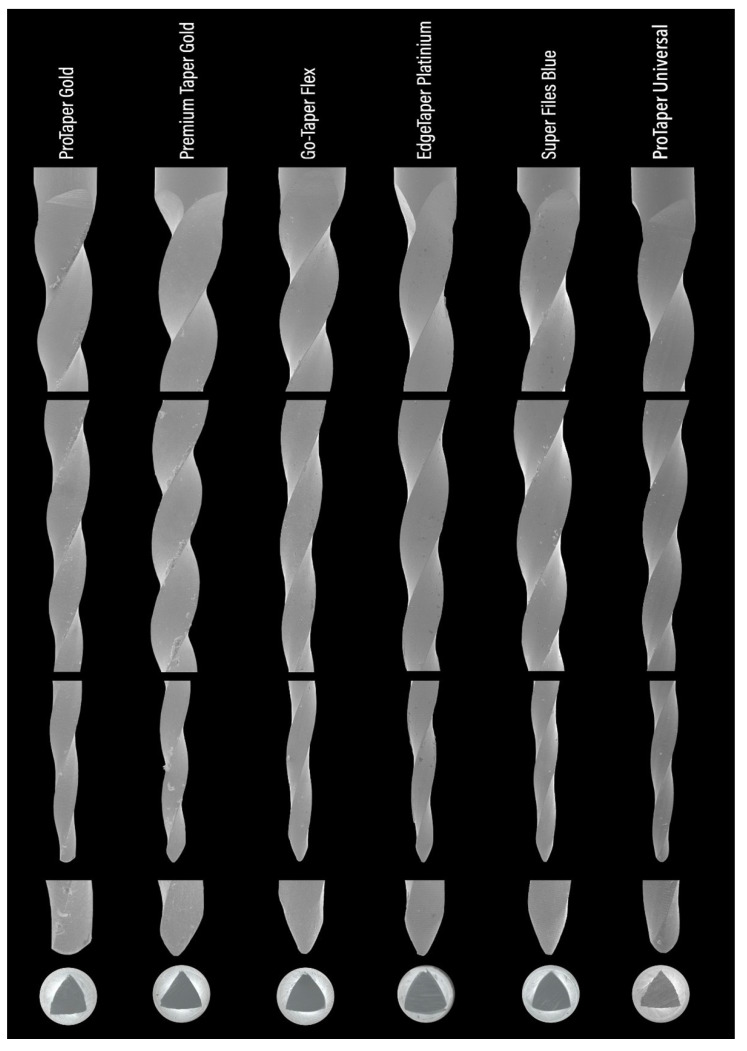
Representative SEM images of the tested instruments showing (from top to bottom) the coronal, middle, and apical portions of the active blades, as well as their tips’ geometry and cross-sectional design. Overall, the instruments’ designs were similar, except for the cross-sectional shape of the Premium Taper Gold instrument and the geometries of the non-active tips.

**Figure 3:**
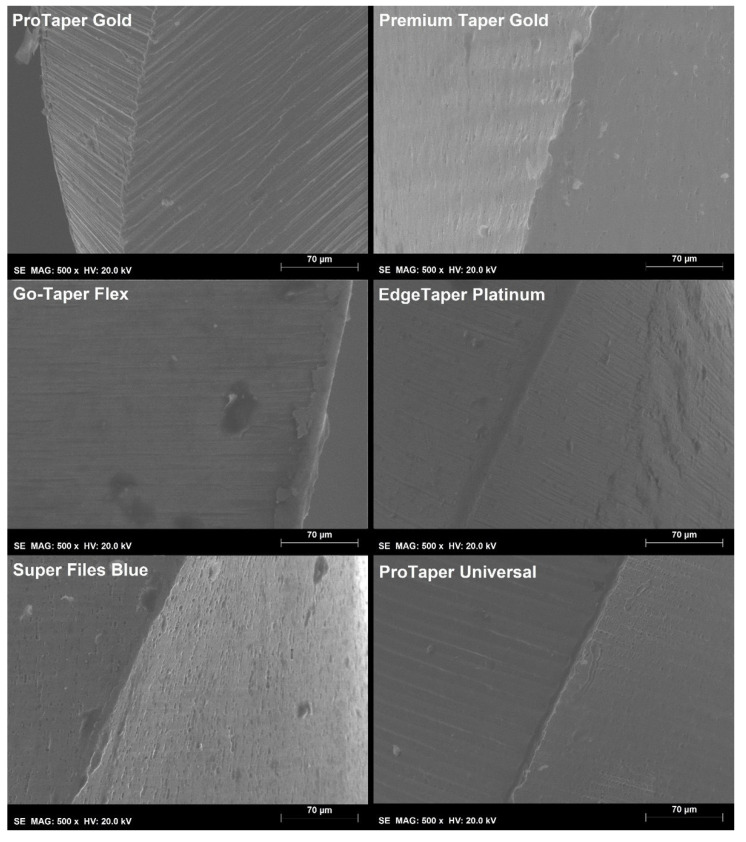
SEM images of the instruments’ surfaces showing distinct marks from the manufacturing process in both ProTaper Gold and ProTaper Universal, porosities of different sizes on the Super Files Blue surface, and less irregularities in the Premium Taper Gold instrument.

The stereomicroscopic analysis (Table 2) revealed similarities between the instruments—except for the Premium Taper Gold instrument, which showed a higher number of blades (n = 15) and median helical angle (29.5°) (*p* < 0.05)—while no major defects, such as missing, twisted, or distorted blades, were observed. On the other hand, none of the alternative instruments showed the position of the 20-mm and 22-mm measuring lines within 0.1 mm of the median values obtained in the premium control brands (PTG and PTU). A SEM analysis (Figure 2) confirmed the design similarities among the instruments with symmetrical spiral designs without radial lands, although the cross-sectional shape of the Premium Taper Gold was more triangular than the convex triangular shape observed in the other instruments. None of the tips could be clearly identified as active and their distance to the transition angle of the blade, as well as its geometry, varied from instrument to instrument. A higher magnification of the active part of the instruments showed a very distinct surface finishing (Figure 3), with both PTG and PTU presenting marks from the manufacturing process on their surface, while the surface of the Super Files Blue had porosities of different sizes. Amongst all instruments, the surface of the Premium Taper Gold instrument displayed the least amount of irregularities. 

### 3.2. Metallurgical Characterization 

**Figure 4:**
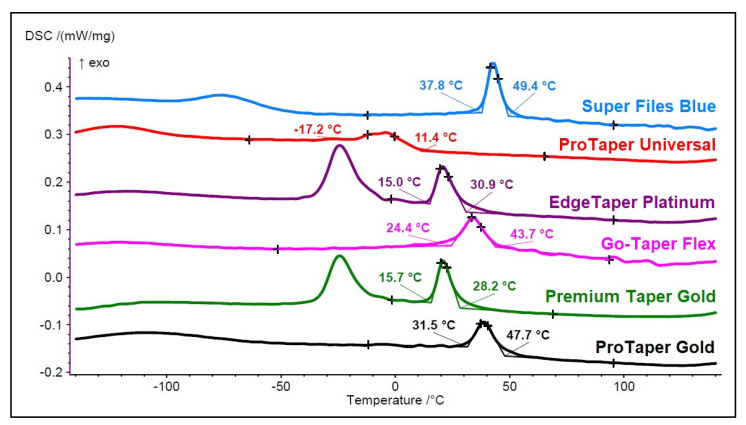
DSC chart shows the cooling curves of the ProTaper Gold (in black), Premium Taper Gold (in dark green), Go-Taper Flex (in pink), EdgeTaper Platinum (in purple), ProTaper Universal (in red), and Super Files Blue (in blue). The chart highlights the R-phase start (Rs) (on the right) and finish (Rf) (on the left) temperatures of each instrument. Overall, the DSC analysis showed differences in the phase transformation temperatures amongst the tested systems, with Rs temperature of heat-treated instruments above 28.2 °C.

In the EDS/SEM analysis, no metal element was detected other than titanium and nickel, with similar Ni/Ti atomic ratios of 1.010 (PTG), 1.030 (Premium Taper Gold), 1.031 (PTU), 1.033 (Go-Taper Flex and Super Files Blue), and 1.039 (EdgeTaper Platinum) found. Assessment of the DSC chart (Figure 4) demonstrated that the closest phase transformation temperatures to the original PTG was achieved by the Go-Taper Flex, while the most distinct were observed with the Premium Taper Gold and EdgeTaper Platinum instruments. On the other hand, Super Files Blue showed the highest R-phase start of the austenite to R-phase transformation during cooling (Rs = 49.4 °C) and finish (Rf = 37.8 °C) temperatures. Conventional NiTi alloy from the PTU instruments had the lowest Rs and the least pronounced transformation “peaks”, with the full austenitic phase occurring above 11.4 °C.

### 3.3. Microhardness 

**Figure 5:**
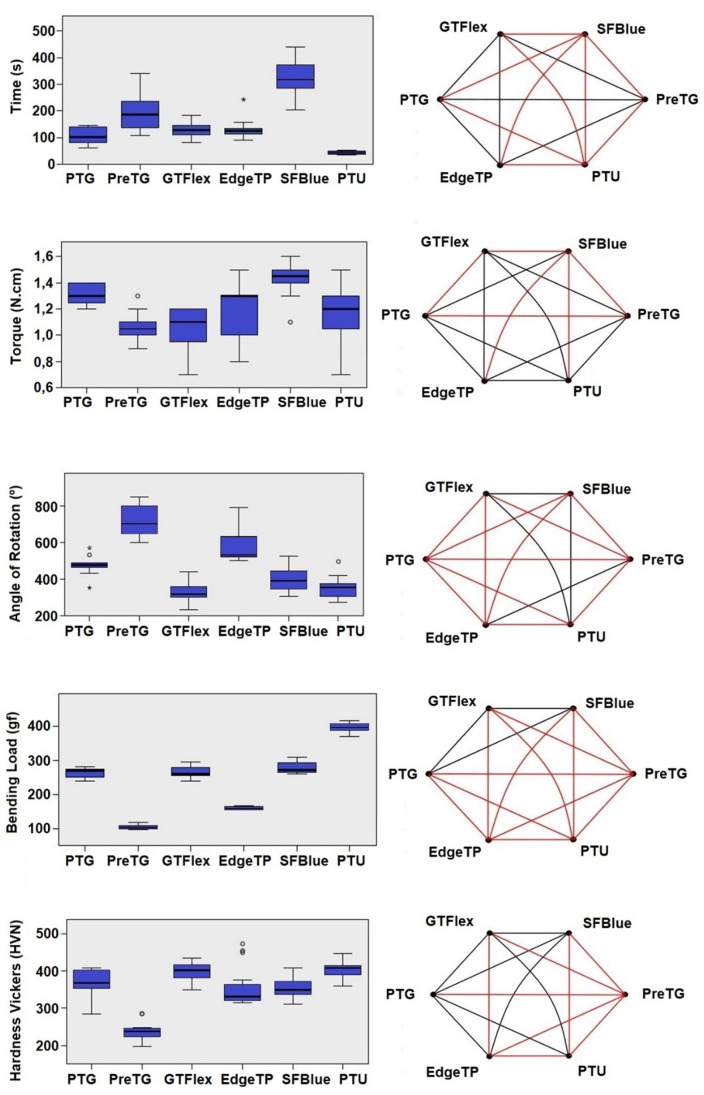
Mechanical performance of the instruments is represented by combined box-and-whisker plots (on the left) of time to fracture, torque, angle of rotation, bending load, and microhardness results, with the line within each box representing the median value. On the right, the diamond graphic details the statistical significance among groups, with the red line representing significant difference (*p* < 0.05) (PTG: ProTaper Gold; PreTG: Premium Taper Gold; GTFlex: Go-Taper Flex; EdgeTP: EdgeTaper Platinum; SFBlue: Super Files Blue; PTU: ProTaper Universal).

Go-Taper Flex and PTU presented the highest microhardness (410.5 HVN and 408.3 HVN, respectively) followed by ProTaper Gold (369.9 HVN) (*p* > 0.05). The lower results were shown by Premium Taper Gold (237.4 HVN), which were significantly different to all other systems (*p* < 0.05) (Table 3, Figure 5).

### 3.4. Mechanical Tests 

Results from the mechanical tests are shown in Table 3 and Figure 5. Super Files Blue showed significantly higher time to fracture (319 s) than all tested instruments (*p* < 0.05), while the lowest median time was observed with PTU (44 s) (*p* < 0.05). No difference was observed in the maximum torque required to fracture between PTG (1.30 N·cm) and the other heat-treated instruments, except for the Premium Taper Gold (1.05 N·cm) and Go-Taper Flex (1.10 N·cm) instruments, which presented significantly lower results (*p* < 0.05). On the other hand, the angle of rotation of the PTG instrument (478°) was significantly different from all other systems (*p* < 0.05). The highest and lowest median angle values were observed with the Premium Taper Gold (702°) and Go-Taper Flex (319°) instruments, respectively. The maximum loads for bending were observed in the Go-Taper Flex (260.6 gf) and Super Files Blue (270.7 gf) instruments, which showed no statistical difference from the PTG (269.2 gf) (*p* > 0.05), while significantly lower values than PTG were observed with the EdgeTaper Platinum (158.3 gf) and Premium Taper Gold (103.5 gf) instruments (*p* < 0.05). The highest load required for bending was observed with PTU (395.1 gf) (*p* < 0.05).

## 4. Discussion

The dental industry has experienced massive growth in recent years, mostly driven by the corporations of major emerging economy countries in East Asia. With the improvement of the global economy by the increasing of e-commerce, a diversity of supplies is readily available to customers worldwide. Although some innovative and original products have been developed and marketed, there are several others that just imitate the physical appearance of well-known brands [8]. With these alternative systems, also known as replica-likes, the consumer is lacking knowledge about their performance and safety. Hence, this study is the first to provide new insights into the mechanical performance of four worldwide and commercially available alternative heat-treated rotary systems from the original PTG brand. Considering the fact that these systems are already being marketed, it becomes urgent that the knowledge of their multiple features and capabilities is known in order to have a comprehensive understanding of their real characteristics. In order to provide an answer to a series of questions, multimethod research was conducted. This mixed approach allowed us to assess aspects such as the manufacturing quality, metallurgical properties, microhardness, and geometric shapes of these systems in order to achieve a better comprehension of the results obtained for the cyclic fatigue, torsional resistance, and bending load tests, using the heat-treated PTG and the conventional PTU systems as controls. In the present study, the tested systems were quite similar regarding their NiTi atomic ratio composition. Stereomicroscopic (Figure 1) and SEM (Figure 2) analyses also confirmed that most of the instruments were similar to PTG regarding the overall design, except for the Premium Taper Gold (Table 2). On the other hand, tip geometry was quite different among all of them (Figure 2). Given this, the first null hypothesis was rejected. 

A macroscopic analysis of the instruments revealed dissimilarities in the color on their surfaces (Figure 1), suggesting potential differences in the metallurgical processing methods employed in their production. In this way, and apart from the PTG and PTU systems, whose NiTi alloys are publicly designated as gold heat treatment [4] and conventional wire [4], respectively, as well as the EdgeTaper Platinum alloy described as heat-treated Firewire [13], no specific information is provided by the other manufacturers—except that their instruments are heat-treated. The heat treatment of the instruments was confirmed by the martensitic–austenitic transformation phases observed in the DSC test. The DSC analysis also showed significant differences in the R-phase start and finish temperatures of the austenite to R-phase transformation during cooling among the systems. Therefore, the second null hypothesis that was tested was rejected. These differences, however, were of the utmost importance for explaining most of the results obtained in the mechanical tests. For example, all of the tested heat-treated instruments showed higher times to fracture and more flexibility than the conventional PTU system (Table 3, Figure 5), supporting previous findings [7,14]. This happened because of the full austenitic composition of the PTU instrument (R-phase start of 11.4 °C) at the testing temperature (20 °C), which required a high stress to induce the martensitic transformation and ultimately reduce the time to fracture and increase the bending load [15] compared to the other instruments, in which the R-phase start temperatures were above 28.2 °C (Figure 4). 

In the cyclic fatigue test, the highest time to fracture amongst all tested instruments was observed with Super Files Blue (319 s) and the lowest one with PTU (44 s) (Table 3). This difference could be explained by the difference between the R-phase start temperature of PTU (11.4 °C) and Super Files Blue (49.4 °C) (Figure 4). On the other hand, PTG and Super Files Blue have very similar R-phase start temperatures (47.7 °C and 49.4 °C, respectively) (Figure 4) but quite different times to fracture (101 s and 319 s, respectively) (Table 3). The higher surface roughness of PTG, compared to that of Super Files Blue (Figure 3), could explain the lower fatigue strength of PTG (101 s) compared to that of Super Files Blue (319 s). Although no detailed information is available about the manufacturing process of Super Files Blue, the heat treatments that turn the instrument’s surface into a bluish color (Figure 1) have been correlated with a significant improvement in cyclic fatigue resistance [3,16,17,18]. As it would be expected, the stiffness of the conventional alloy of the PTU system resulted in the lowest cyclic fatigue resistance, which is in agreement with the previous studies [5,14,19]. On the other hand, the cyclic fatigue results of the other systems were similar to PTG (Figure 5). One of the possible reasons that might help to explain the similarity of Go-Taper Flex and PTG can be their closer transformation temperatures (24.4–43.7 °C and 31.5–47.7 °C, respectively), as observed in the DSC test (Figure 4), while EdgeTaper Platinum may be related to the experimental setup used in this study. According to the manufacturer, EdgeTaper Platinum would have “twice the cyclic fatigue as ProTaper Gold and six times that of ProTaper” [20]. These apparently contradictory results might be justified because of methodological differences. Although both studies used artificial canals with severe curvature, the manufacturer’s setup consisted of inserting 3 mm of the tip into a test block with a 90° curvature, while in the present study the instruments rotated freely on a 9-mm length of stainless-steel non-tapered artificial canal (6 mm radius and 86 degrees of curvature) with the position of maximum stress located at the middle of the curvature length. Finally, although no statistical difference was observed between the time to fracture of the Premium Taper Gold (186 s) and PTG (101 s) instruments, the high median value of the former is possibly a consequence not only of its better surface finishing (Figure 3) [21], but also due to its small cross-sectional core size (Figure 2), high helical angle, and number of blades (Table 2) [22]. 

The failure of NiTi instruments is a frequent topic of discussion, and torsional overload is the most common type of fracture during canal shaping [23]. Torsional strength indicates the enhanced ability of an instrument to twist before fracture, which is needed in the preparation of narrow and constricted canals when the instrument is exposed to high torsional loads [24]. Overall, it would be expected that instruments that require higher torque to fracture during a torsional test would have less flexibility and less cyclic fatigue resistance [6,22]. However, several factors could substantially affect the torsional resistance of the NiTi rotary instruments, including the cross-sectional design, chemical composition of the alloy, and thermomechanical process applied during manufacturing [7,25,26]. In the present study, the maximum torque for fracture of PTG, EdgeTaper Platinum, and Super Files Blue showed no difference from the conventional PTU. Despite the clear differences in their transformation temperatures, a similar torsional resistance of PTG and PTU has already been reported in other studies using the same methodology as here [19,27]. Similarly, other reports comparing conventional and heat-treated NiTi instruments also demonstrated a higher cyclic fatigue strength for the former, but no difference regarding the torsional strength [28,29]. Thus, although it was also observed in this study that alterations in the manufacturing process enhanced the cyclic fatigue resistance of the heat-treated instruments compared to the PTU system, it did not improve their torsional behavior. Interestingly, this may be attributed to the heat treatment process as well, which preserved the martensitic phase of the instruments at the test temperature [5], allowing them to have a greater amount of deformation than the conventional NiTi alloy until failure [25,27]. This also explains the different angular deflections before fracture observed among them (Figure 5), although they did not correlate with their corresponding torque at failure values, which is in agreement with previous reports [30]. On the other hand, despite a high angular deflection that could be beneficial to prevent instrument separation, it may not have clinical significance considering that a complete rotation occurs in 0.2 s at a speed of 300 rpm [30,31]. In the torsional test, it would be expected that there would be significant differences in the maximum torque to failure between Super Files Blue and PTG, considering their distinct metallurgical properties (Figure 4). However, the torsional testing performed in this study does not provide a suitable condition to evaluate the effect of the overall geometry of the instruments, rather, it provides their behavior only at the area where the instrument was held. In this way, additional morphometric measurements of the overall dimensions at the tip of Super Files Blue showed that it was at least 15–20% larger than PTG (Figure 1). Given this, the increase in size is compensated for by its higher transformation phase temperatures, thereby explaining the results. The other two heat-treated systems (Premium Taper Gold and Go-Taper Flex) had lower median torque values than PTG. The results of Premium Taper Gold can be explained by its design, with its high number of blades per unit length and large helical angles [22], while Go-Taper Flex is not clear. It is possible, however, to infer that the high surface finishing quality of Go-Taper Flex (Figure 3) associated with other aspects related to its design, such as the core diameter, taper, and/or percentage of contaminants added to the alloy during the manufacturer process, which were not assessed in this study, had affected the result. 

In the bending test, Premium Taper Gold and Edge Taper Platinum showed the lowest bending loads and Go-Taper and Super Files Blue performed similarly to PTG while, as expected, the conventional PTU instrument had a significantly higher median value [32,33] (Table 3). The increased flexibility of Premium Taper Gold could be explained by its high number of blades and large helical angle [22], and Edge Taper Platinum as a consequence of its smaller dimensions. According to the manufacturer, the F1 Edge Taper Platinum is a size 20 constant 0.06 tapered instrument [13], whilst the PTG F1 has a 0.07 taper in its first 3 mm that progressively decreases towards the end of the cutting blade [34]. On the other hand, one of the reasons that might help to explain the similarity in the bending load results of Go-Taper Flex and PTG might be their close R-phase transformation temperatures and, although the result of Super Files Blue seemed contradictory considering its highest R-phase temperatures (Figure 4), the large dimensions of its tip, which, as previously mentioned, justify the observed results. Therefore, the differences in the mechanical properties of the tested instruments lead to the rejection of the fourth null hypothesis. 

It has been mentioned that a superior surface microhardness may lead to a superior cutting efficacy considering that dentine microhardness is only around 67 HVN [24,32]. In the present study, the PTU conventional alloy presented amongst the highest hardness Vickers numbers (408.3 HVN), which corroborates with previous studies [24,32]. The lower results from Premium Taper Gold (237.4 HVN) may anticipate more difficulties in the cutting capacity of this system. Considering the differences observed amongst the instruments, the third null hypothesis was also rejected. The present results show important differences between the heat-treated premium brand and some replica-like systems, a condition which corroborates with a similar previous study that also assessed F1 instruments that used conventional NiTi alloys [35]. 

In this study, a DSC analysis and torsional and bending resistance tests were performed according to international standards [10,12], and little debate exists regarding their methodological processes regardless of small variations that may have also already been used [25,36]. In contrast, no specification is available for the cyclic fatigue test and, throughout the years, successive modifications have been proposed to the method in an attempt to mimic the clinical settings [37]. These variations, however, have been considered its major drawback [37] since the outcomes from different studies may be unfeasible to compare. Although it is true that performing the cyclic fatigue test under the same experimental conditions would allow for the control of the interference of several confounding variables, thereby increasing its internal validity [38]. On the other hand, several breakthrough findings would never have been reached if it would not be possible to vary a particular setting in order to evaluate its influence on the final outcome. The present test settings have been previously published [8,35,39] and have included the static model, which has been associated with a higher internal validity compared to the dynamic model [37], and the use of room temperature that is in accordance with an international guideline for conducting tension tests on NiTi super-elastic materials, which mentions testing at 22 °C ± 2 °C [40] unless otherwise specified. Even though, in order to assess the possible influence of the test temperature in the outcomes, a differential scanning calorimetry analysis was conducted, which allows an interpretation of the possible variations in the entire service temperature range depending on the temperature at which the phase transformations occur on each instrument. Further research should be directed at other lesser known but globally available instruments, a superior assessment of the instruments’ real dimensions and design, and the real manufacturing heat treatment procedures of these instruments.

## 5. Conclusions

Although similarities in the overall design and atomic ratio elements were not between instruments, differences in the surface finishing and phase transformation temperatures tended to influence their overall mechanical performances. Super Files Blue outperformed PTG in the cyclic fatigue test while EdgeTaper Platinum and Premium Taper Gold showed higher flexibility in the bending test. On the other hand, most systems had similar torque to fracture compared to the PTG, except for Premium Taper Gold and Go-Taper Flex that showed lower results. Go-Taper Flex and PTU showed superior surface microhardness. Taking into consideration the observed differences, the clinicians might expect different outcomes in clinical practice.

## Figures and Tables

**Table 1:** Characteristics of the six tested rotary NiTi systems.

System	Corresponding NiTi Metal Alloy	Manufacturer Specifications	Identification (Color Coding)	Lot
ProTaper Gold	Thermo-treated	Dentsply (Ballaigues, Switzerland)	(A)	1523909
Premium Taper Gold	Thermo-treated	Waldent (City not stated, China)	(A)	201808
Go-Taper Flex	Thermo-treated	Access (Shenzhen, China)	(B)	17110103
EdgeTaper Platinum	Thermo-treated	EdgeEndo (Johnson City, TN, USA)	(A)	070717008
Super Files Blue	Thermo-treated	Flydent (Shenzhen, China)	(A)	Not available
ProTaper Universal	Conventional	Dentsply (Ballaigues, Switzerland)	(A)	1032529

(A) Full set of intruments: SX (none), S1 (pink), S2 (white), F1 (yellow), F2 (red), F3 (blue). (B) Full set of intruments: A0 (none), A1 (pink), A2 (white), B1 (yellow), B2 (red), B3 (blue).

**Table 2:** Stereomicroscopic assessment of instruments (median and interquartile range).

NiTi Instrument	n	Number of Blades ^1^	Helical Angle (°) ^1^	Measuring Lines Position (in mm) ^2^		
				18 mm	20 mm	22 mm
ProTaper Gold F1	6	12	25.0 [24.0–25.3]	18.01 [17.97–18.08]	20.02 [19.98–20.11]	21.96 [21.92–22.01]
Premium Taper Gold F1	6	**15**	**29.5 [28.8–30.3]**	**18.15 [18.12–18.27]**	**20.23 [20.09–20.37]**	**22.18 [22.09–22.55]**
Go-Taper Flex B1	6	12	24.0 [23.8–25.3]	18.05 [17.89–18.23]	**20.27 [20.03–20.36]**	**22.25 [21.89–22.34]**
EdgeTaper Platinum F1	6	12	25.0 [23.8–25.0]	**18.22 [18.05–18.41]**	**20.35 [20.23–20.52]**	**22.16 [21.93–22.35]**
Super Files Blue F1	6	12	25.5 [23.8–26.0]	**18.21 [18.12–18.37]**	**20.15 [19.96–20.26]**	**22.34 [22.17–22.45]**
ProTaper Universal F1	6	12	25.5 [24.8–26.0]	18.06 [18.01–18.17]	19.97 [19.92–20.07]	22.06 [21.99–22.16]

^1^ Values in bold letters mean statistical significant difference in the same column (*p* < 0.05). ^2^ Significant discrepancies in the mean measuring line positions were identified with bold letters when values were higher than 0.1 mm from the reference value.

**Table 3:** Median (interquartile range) results of the mechanical tests and microhardness *.

NiTi Instrument	Cyclic Fatigue		Torsional Resistance		Bending Resistance	Microhardness
	Time to Fracture (s)	Fragment Length (mm)	Maximum Torque (N·cm)	Angle of Rotation (°)	Maximum Load (gf)	Hardness (HVN)
ProTaper Gold F1	101.5 [81.5–141.8]	7.4 [6.8–7.9]	1.30 [1.23–1.40]	478 [462–490]	269.2 [249.9–274.8]	369.0 [351.2–402.7]
Premium Taper Gold F1	186.0 [131.8–238.8]	7.8 [7.5–7.9]	1.05 [1.00–1.10]	702 [643–803]	103.5 [99.70–107.7]	237.4 [220.9–245.7]
Go-Taper Flex B1	128.5 [107.5–148.8]	7.8 [7.3–8.2]	1.10 [0.93–1.20]	319 [298–361]	260.6 [253.4–279.4]	410.5 [401.5–427.8]
EdgeTaper Platinum F1	125.0 [113.3–136.3]	7.2 [6.9–7.7]	1.30 [0.95–1.30]	535 [519–669]	158.3 [155.1–164.7]	332.6 [320.6–376.4]
Super Files Blue F1	319.0 [283.5–376.3]	6.6 [5.8–7.7]	1.45 [1.40–1.50]	393 [342–449]	270.7 [263.7–292.9]	349.8 [334.7–378.0]
ProTaper Universal F1	43.1 [37.0–50.5]	7.8 [7.7–7.9]	1.22 [1.18–1.31]	356 [306–381]	397.1 [386.9–408.3]	408.3 [387.6–415.9]

* Figure 5 summarizes the statistical differences among tested systems.
